# Negative fluid balance 48 hours after admission improves survival at 28 days in critically ill patients

**DOI:** 10.1186/cc10848

**Published:** 2012-03-20

**Authors:** M Cuartero, AJ Betbese, K Nuñez, J Baldira, L Zapata

**Affiliations:** 1 Hospital De Sant Pau, Barcelona, Spain

## Introduction

Fluid infusion may be lifesaving in critically ill patients, but following initial resuscitation a positive fluid balance is associated with increased mortality. This study aimed to determine whether a negative fluid balance (≤-500 ml) within the first 48 hours of admission in the ICU is associated with improved survival at 28 days in a heterogeneous cohort of critically ill patients.

## Methods

We conducted a retrospective study in a 20-bed ICU at a university-affiliated teaching hospital. Patients admitted for acute heart failure, those who required dialysis before admission and those who died within 24 hours after admission were excluded. Demographic data, SAPS II and APACHE II scores were recorded at admission and SOFA, fluid balance, hemodynamic, respiratory and renal variables once per day. Variables were compared between survivors and nonsurvivors and between patients who did and those who did not achieve negative fluid balance by day 2 of admission. Multiple logistic regression was used to identify variables significantly associated with ICU mortality in the univariate analysis. Survival was assessed using Kaplan-Meier analysis.

## Results

We studied 87 patients: 53 males, mean age 58 ± 18 years, SAPS II 39.3 ± 15.8, APACHE II score 15.9 ± 7.5, SOFA score 5.0 ± 3.4, and ICU stay 10.3 ± 9.8 days. The main syndrome diagnosis at admission was septic shock (*n *= 26), acute respiratory failure (*n *= 19), trauma (*n *= 13), neurocritical illness (*n *= 14) and others (*n *= 15). Overall mortality in the ICU reached 20.7% and survival at 28 days was 73.6%. When patients were classified according to 28-day outcome, we observed statistically significant differences in negative fluid balance at 48 hours (*P *< 0.001), SAPS II (*P *< 0.001), APACHE II score (*P *= 0.007), age (*P *= 0.046) and incidence of acute kidney injury at admission (*P *= 0.02; defined as at least Risk in RIFLE criteria), but urinary output, hemodynamic and respiratory parameters did not differ. Multivariate analysis showed that negative fluid balance at 48 hours was independently associated with improved survival: odds ratio = 7.9 (*P *= 0.013). Kaplan-Meier analysis showed that survival was significantly lower in patients without negative fluid balance at 48 hours (*P *= 0.015).

## Conclusion

Our findings show that negative fluid balance 48 hours after admission may correlate with better outcome in a heterogeneous population of critically ill patients.
